# In Situ Hybridization Strategy Constructs Heterogeneous Interfaces to Form Electronically Modulated MoS_2_/FeS_2_ as the Anode for High-Performance Lithium-Ion Storage

**DOI:** 10.3390/molecules29061387

**Published:** 2024-03-20

**Authors:** Dazhi Li, Changlong Sun, Zeqing Miao, Kesheng Gao, Zeyang Li, Wei Sun, Shengjing Guan, Xiaofei Qu, Zhenjiang Li

**Affiliations:** 1 College of Materials Science and Engineering, Qingdao University of Science and Technology, Qingdao 266042, China; wslidazhi@163.com (D.L.); changlongsun@qust.edu.cn (C.S.); m2043714499@126.com (Z.L.); weisun098@163.com (W.S.); 2 College of Electromechanical Engineering, Qingdao University of Science and Technology, Qingdao 266061, China; happymiaozeqing@163.com; 3 Songshan Lake Materials Laboratory, Dongguan 523808, China; gaokesheng2023@163.com; 4 School of Chemistry and Chemical Engineering, Shandong University of Technology, Zibo 255049, China; guanshengjing2022@126.com

**Keywords:** MoS_2_/FeS_2_, heterojunction, interfacial effect, electronically modulate

## Abstract

The interfacial effect is important for anodes of transition metal dichalcogenides (TMDs) to achieve superior lithium-ion storage performance. In this paper, a MoS_2_/FeS_2_ heterojunction is synthesized by a simple hydrothermal reaction to construct the interface effect, and the heterostructure introduces an inherent electric field that accelerates the de-embedding process of lithium ions, improves the electron transfer capability, and effectively mitigates volume expansion. XPS analysis confirms evident chemical interaction between MoS_2_ and FeS_2_ via an interfacial covalent bond (Mo–S–Fe). This MoS_2_/FeS_2_ anode shows a distinct interfacial effect for efficient interatomic electron migration. The electrochemical performance demonstrated that the discharge capacity can reach up to 1217.8 mA h g^−1^ at 0.1 A g^−1^ after 200 cycles, with a capacity retention rate of 72.9%. After 2000 cycles, the capacity retention is about 61.6% at 1.0 A g^−1^, and the discharge capacity can still reach 638.9 mA h g^−1^. Electrochemical kinetic analysis indicated an enhanced pseudocapacitance contribution and that the MoS_2_/FeS_2_ had sufficient adsorption of lithium ions. This paper therefore argues that this interfacial engineering is an effective solution for designing sulfide-based anodes with good electrochemical properties.

## 1. Introduction

Lithium-ion batteries (LIBs) garner extensive attention as energy storage devices, owing to their stable voltage platform and outstanding energy density [1,2]. However, the unsatisfactory discharge capacity, charging time, and cycle life of the graphite anode seriously limit the widespread application of LIBs [3]. Therefore, substitutable anodes with higher structure stability and practical capacity have been widely sought after in the last few years [4,5]. TMDs generally have the advantages of higher theoretical capacity and lower reaction potential, which makes them a kind of anode material with excellent prospects for development. Among them, MoS_2_ has received extensive attention from researchers as an anode material for lithium-ion batteries with prospects for industrial applications due to its high theoretical capacity (670 mA h g^−1^) and simple preparation process. According to a previous report, the high capacity of MoS_2_ is achieved via lithium-ion insertion (MoS_2_ + xLi^+^ + xe^−^ ↔ Li_x_MoS_2_) and conversion reaction (Li_x_MoS_2_ + (4 − x) Li^+^ + (4 − x) e^−^ ↔ Mo + 2Li_2_S) [6]. The formation of Li_2_S, however, results in the dissolution of Li_2_S into the liquid electrolyte and disrupts the electrical contact between the TMDs and the current collector. Consequently, this phenomenon significantly compromises cycling stability and induces excessive capacity decay during cycling. The interstitial of the MoS_2_ lattice can be adjusted via structural design and phase transition engineering to control the amount of lithium-ion insertion, thus realizing the purpose of controlling the capacity. Meanwhile, the good structural stability guarantees its stable lithium-ion storage performance [7]. Therefore, MoS_2_-based anodes attract significant attention for high-efficiency lithium-ion storage, with potential application prospects [8]. However, the MoS_2_-based anode still suffers from low electronic conductivity, unsatisfied lithium-ion transport kinetics, and large volume expansion. As a result, the MoS_2_-based anode exhibits limited capability under the long-term charge/discharge process and rate capability. Moreover, the solid electrolyte interphase (SEI) film can give rise to “dead lithium” and unstable Coulombic efficiency (CE) [9].

To address the above issues, morphological modification, structural optimization, and vacancy design are adopted [10]. Jiao et al. reported an outer-wall-free hollow nanotube composed of intersecting small-sized MoS_2_ nanosheets. The metal nanotubes are hierarchical, hollow, and have a porous arrangement, which aid electrolyte transport and diffusion, avoid the re-stacking of 2D nanosheets, and shorten the ionic diffusion path, thus maintaining the stability of electrochemical cycling and improving the rate performance [11]. Chen et al. prepared nanoflowers crossed by MoS_2_-MnS heterojunction nanosheets, confirming the phenomenon of sequential phase transition of MoS_2_ and MnS in Li/Na storage, and electrochemical reaction kinetics and ex situ tests confirmed the roles of phase transition and interfacial engineering in the reaction [8]. Bai et al. prepared 1T-MoS_2_/C by hydrothermal reaction and confirmed that 1T phase-transformed MoS_2_ has a high conductivity characteristic due to the nature of the metallic phase, which facilitates rapid transport of lithium ions and electrons and thus improves the electrochemical performance [12]. However, the design reported above mainly focuses on the exterior morphology control, electrochemical conductivity, and reaction kinetics of the reported MoS_2_ anode, which are inferior to those of the graphite anode, and the volume change is not fully released during the electrochemical reaction. It is unclear whether good adsorption ability toward lithium ions, good conductivity for charge transfer, and conversion kinetics are achieved simultaneously in those reported MoS_2_ anodes. Moreover, the inadequate interfacial interaction in MoS_2_-based anodes further influences the electrochemical conductivity and reaction kinetics [13]. Therefore, the reported lithium-ion storage performance of MoS_2_ anodes is inferior, and constructing a multi-advantage MoS_2_ anode is highly required.

An interfacial engineering strategy is considered as an effective route to accelerate the charge transfer kinetics, adsorption, and intercalation ability of lithium-ion batteries via a strong interfacial bonding effect at the interface to achieve high-performance lithium-ion storage [14,15]. Moreover, the interfacial design can create sufficient active sites at the interface, which is significant for excellent lithium-ion storage capacity. However, an interfacial engineering strategy has rarely been reported to construct MoS_2_ anodes, and the internal mechanism of interfacial interaction remains unclear. FeS_2_ has the advantages of high chemical stability and high theoretical capacity (890 mA h g^−1^), which makes it a satisfactory candidate for constructing MoS_2_-based composites [16]. Its incorporation enhances the stability of the material and improves the ionic conductivity and kinetic behavior of the MoS_2_ anode. Sufficient interfacial contact between MoS_2_ and FeS_2_ would provide abundant interfacial sites to adsorb lithium ions. After the interfacial design, the interfacial region can adsorb a great deal of lithium ions and guarantee high electron transfer efficiency [17]. As a result, the lithium-ion storage capacity and reaction kinetics of MoS_2_/FeS_2_ are prominently enhanced. However, constructing interfacial regions and enhancing the lithium-ion storage capacity of MoS_2_/FeS_2_ have seldom been researched so far.

Herein, phase transition engineering and interfacial engineering strategies are adopted to construct the MoS_2_/FeS_2_ anode. The interfacial covalent bond (Mo–S–Fe) is confirmed by TEM and XPS analyses. Enhanced pseudocapacitance contribution and improved charge transfer efficiency are further revealed by electrochemical kinetic analysis. Consequently, this well-designed MoS_2_/FeS_2_ anode demonstrates excellent cycling capacity, rate capability, and cycling life in half cells. This interfacial design strategy can enhance lithium-ion storage performance via the combined impact of surface sulfide and interfacial interaction, as well as provide a deeper understanding of re-designing traditional metal sulfide anodes [18].

## 2. Results

**Figure 1:**
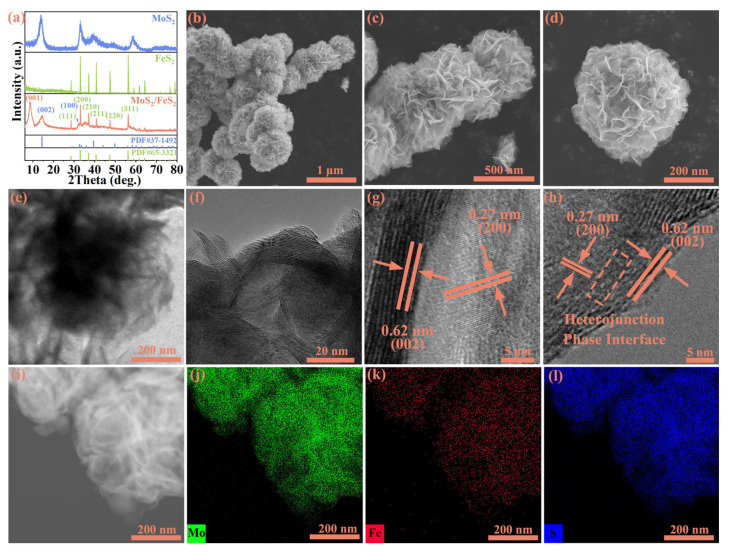
(**a**) XRD patterns of MoS_2_/FeS_2_, pure MoS_2_, and FeS_2_. (**b**–**d**) SEM of MoS_2_/FeS_2_ nanoflower spheres. (**e**–**h**) TEM and HRTEM of MoS_2_/FeS_2_ nanoflower spheres, respectively. (**i**–**l**) TEM (inset) and elemental mapping analysis of Mo, Fe, and S in MoS_2_/FeS_2_ nanoflower spheres, respectively.

MoS_2_/FeS_2_ nanoflower spheres are synthesized by a simple and low-cost hydrothermal reaction. More details about the synthetic process can be found in the Materials and Methods section. During the hydrothermal reaction, the transition from 2H-MoS_2_ to 1T- MoS_2_ in some MoS_2_ is induced by a change in coordination modes between certain molybdenum and sulfur atoms. In Figure 1a, the XRD results reveal that the diffraction peaks of pure MoS_2_ correspond to MoS_2_ (PDF#37-1492), and the diffraction peaks of pure FeS_2_ correspond to FeS_2_ (PDF#65-3321). For MoS_2_/FeS_2_, the characteristic peaks at 14.38°, 39.54°, and 49.79° correspond to the (002), (103), and (105) planes of MoS_2_, and the characteristic peaks at 33.03°, 37.07°, 47.41°, and 56.26°correspond to the (200), (210), (220), and (311) planes of FeS_2_. In addition to this, the diffraction peak appearing at about 9° is widely regarded as a characteristic peak of 1T-phase MoS_2_, obtained by the transformation of part of the 2H-phase MoS_2_ during heterojunction formation [19,20,21]. The XRD results reveal that the hydrothermal reaction successfully constructed MoS_2_/FeS_2_ nanoparticles. SEM is performed to demonstrate the microstructure morphology before and after hydrothermal reaction (Figure S1). The SEM results (Figure 1b–d) confirm that the MoS_2_/FeS_2_ nanoflower spheres are composed of intercrossed small-sized nanosheets, with an average dimension of around 200 nm. The morphology of pure MoS_2_ is irregular particles, and pure FeS_2_ is a plate strip nanosheet (Figure S2). The nanoflower spheres exhibit significantly enlarged specific surface area, facilitating the presence of more active sites and promoting the adsorption and rapid migration of lithium ions. Moreover, interlocking nanosheets contribute to maintaining the structural stability of the material. The rough surface of MoS_2_/FeS_2_ nanoflower spheres is confirmed, and the rough surface and stable chemical characteristics can improve the reversible conversion kinetics and lithium-ion adsorption capacity of MoS_2_/FeS_2_ anodes.

TEM is conducted to demonstrate the presence of an interfacial structure between MoS_2_ and FeS_2_. In Figure 1e, the MoS_2_/FeS_2_ nanoflower spheres are clearly visible with irregular ball-like morphology in the low-magnification TEM image, and the rough surface is crucial for interfacial interaction and lithium-ion storage. The size of the grains measures approximately 200 nm, which aligns with the findings from Figure 1d in the SEM analysis. In Figure 1f, the presence of distinct boundaries between the MoS_2_ and FeS_2_ lattice fringes indicates simultaneous execution of the vulcanization process during hydrothermal reaction [22]. As marked by the orange rectangular box, distinct heterointerface structure regions exist between MoS_2_ and FeS_2_, and this sufficient heterointerface structure shows efficient interatomic electron migration via a covalent chemical bond interaction to improve interfacial charge transfer kinetics and lithium-ion storage performance. The lattice spacing of about 0.27 nm in the figure is the (200) plane of FeS_2_, and the lattice spacing of 0.62 nm is the (002) plane of MoS_2_. The intact and distinct lattices both of MoS_2_ and FeS_2_ indicate that the crystallinity of MoS_2_/FeS_2_ obtained through hydrothermal reaction is excellent (Figure 1g,h). The corresponding Selected Area Electron Diffraction (SAED) further proves the existence of MoS_2_ and FeS_2_ (Figure S3). Thus, strong electrostatic attraction is formed via MoS_2_, and FeS_2_ is generated simultaneously after a hydrothermal reaction. The reconstructed interfacial layer can offer sufficient active reaction sites for lithium-ion adsorption, and it is beneficial for high electronic conductivity and fast electrochemical reaction kinetics [23]. The elemental mapping analysis (Figure 1i–l) shows the rectangular distribution of Mo (green), Fe (red), and S (blue) in MoS_2_/FeS_2_. The intimate contact interface between MoS_2_ and FeS_2_ significantly optimizes the lithium-ion transport path and enhances lithium-ion storage performance [24].

**Figure 2:**
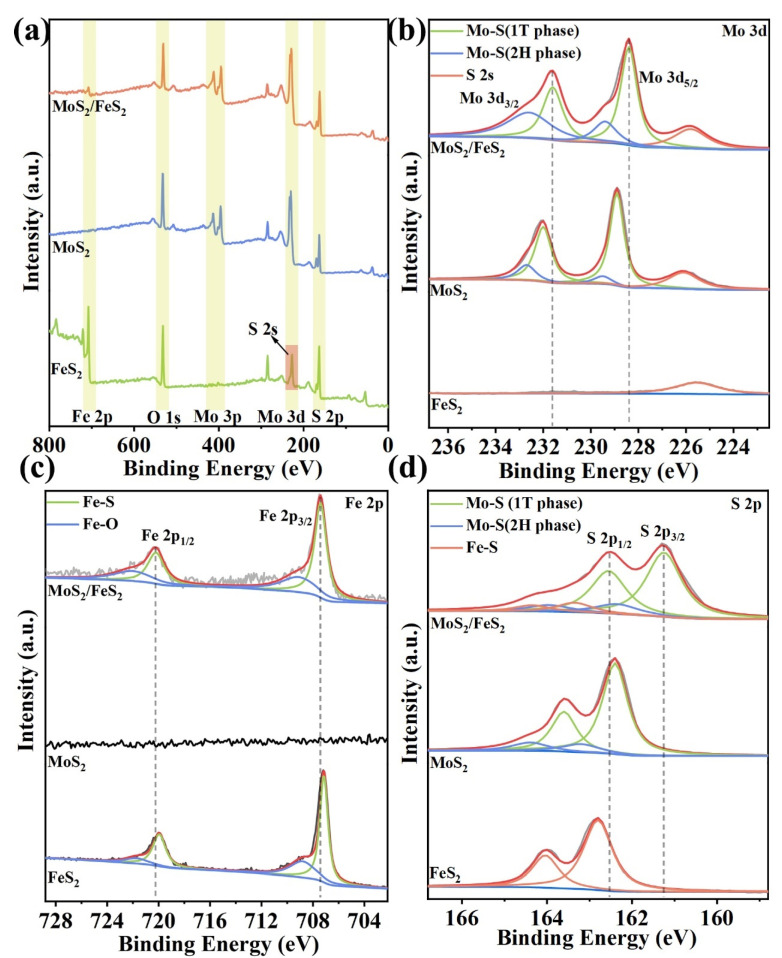
XPS of (**a**) survey spectra, (**b**) Mo 3d, (**c**) Fe 2p, and (**d**) S 2s of MoS_2_/FeS_2_, pure MoS_2_, and FeS_2_, respectively.

The interfacial interaction and chemical composition of MoS_2_/FeS_2_, pure MoS_2,_ and FeS_2_ are further analyzed by XPS. Indeed, Mo 3d, Fe 2p, and S 2p are detected in the survey spectrum of MoS_2_/FeS_2_ (Figure 2a). Only Mo 3d and S 2p are detected in the survey spectrum of MoS_2_. Similarly, Mo 3d is not detected in the survey spectrum of FeS_2_. For FeS_2_, the peak at 225.6 eV in the survey spectrum is S 2s instead of Mo 3d [25]. In Figure 2b, the high-resolution Mo 3d spectra of MoS_2_/FeS_2_ show that the peaks at 228.4 and 231.6 eV belong to Mo 3d_5/2_ and Mo 3d_3/2_ of 1T-MoS_2_, respectively, while the peaks in the 2H phase are located at 229.4 and 232.6 eV, respectively [26]. Compared with pure MoS_2_, the peak of MoS_2_/FeS_2_ is shifted toward low binding energy, and distinct electrostatic attraction is formed with the formation of an efficient electron transport path [18]. The good electronic conductivity of MoS_2_/FeS_2_ is favorable for high reaction kinetics. Therefore, this result shows the evident chemical interaction and strong imbalanced charge distribution between MoS_2_ and FeS_2_, and the distinct interfacial contact provides sufficient interfacial sites for lithium-ion storage [27]. The chemical state of Fe in MoS_2_/FeS_2_ is further analyzed. In Figure 2c, the high-resolution Fe 2p spectrum can be deconvoluted to the Fe–S bond (Fe 2p _3/2_ and Fe 2p _1/2_) at 707.4 and 720.2 eV, respectively. In addition, the high-resolution Fe 2p spectrum also exhibits an obvious peak of Fe combined with O with the formation of a Fe–O bond at 709.2 and 722.2 eV, which is widely regarded as unavoidable oxidation of the lesser samples [28]. Compared to MoS_2_/FeS_2_ with pure FeS_2_, a higher binding energy shift for the Fe–S bond is clearly observed.

Similarly, the S 2p high-resolution spectrum of MoS_2_/FeS_2_ (Figure 2d) exhibits two distinct sets of peaks associated with the Mo–S bond. Specifically, the peaks observed at 161.3 eV and 162.6 eV correspond to the 1T phase, while those detected at 162.4 eV and 164.0 eV are indicative of the presence of the 2H phase. In addition, the remaining set of peaks belonging to Fe–S can be deconvoluted to 163.4 eV and 164.4 eV, respectively. Compared with pure MoS_2_ and pure FeS_2_, all the peaks corresponding to Mo–S bonds move to the direction of low binding energy, while all the peaks corresponding to Fe–S bonds move to the direction of high binding energy, and this phenomenon fully confirms the process of electron transfer from FeS_2_ to MoS_2_. Widespread Mo–S bonds and Fe–S in MoS_2_/FeS_2_ provide good electron conductivity and Faradaic pseudocapacitance. Furthermore, the heterogeneous interface leads to a lopsided charge distribution near the interface, and the corresponding interfacial interaction can boost the electron transfer reactions and offer sufficient active reaction sites for lithium-ion storage. The changes in the Mo–S and Fe–S binding energies demonstrate the interaction between MoS_2_ and FeS_2_, which further enhances the adsorption of lithium ions at the interface. Therefore, the XPS analysis confirms that MoS_2_ and FeS_2_ were formed synchronously during the hydrothermal reaction. The imbalanced charge distribution occurring in the interfacial region can create an inner electric field [29], and the partial phase transition produces 1T-MoS_2_ with metallic conductor properties favoring the electronic conductivity of the MoS_2_/FeS_2_. Moreover, the heterogeneous interface demonstrates strong covalent chemical bond interaction between the MoS_2_ layer and FeS_2_ layer, which is important for interfacial lithium-ion storage. The XPS analysis unambiguously shows that heterogeneous interfaces are formed through the simultaneous generation of the hydrothermal reaction MoS_2_ and FeS_2_ and that heterogeneous interfaces enhance interfacial charge migration for lithium-ion storage.

**Figure 3:**
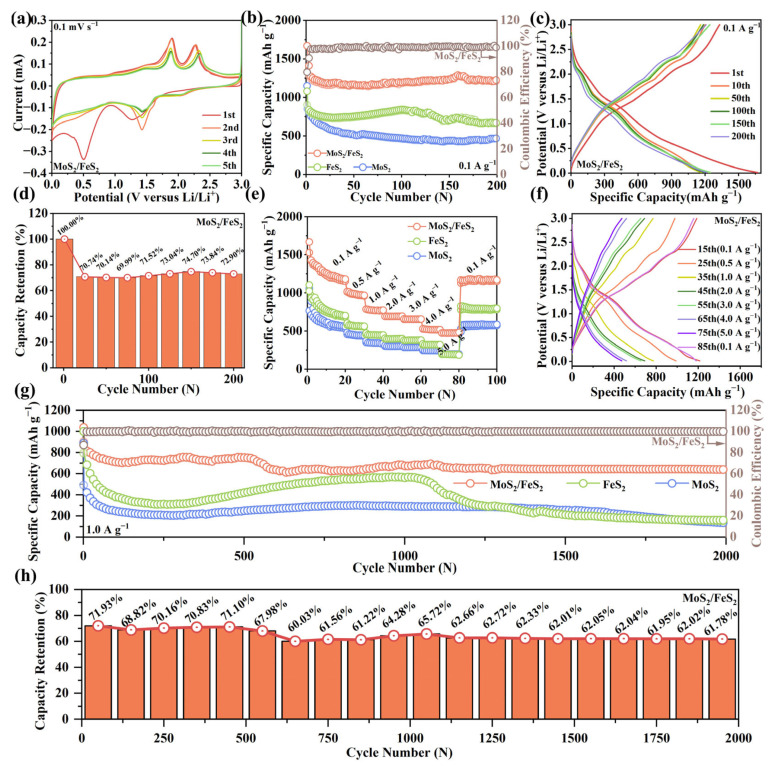
(**a**) Cyclic voltammetry (CV) curves at 0.1 mV s^−1^. (**b**) Cycling performance of MoS_2_/FeS_2_, pure MoS_2_, and FeS_2_ at 0.1 A g^−1^, and Coulombic efficiency of MoS_2_/FeS_2_ at 0.1 A g^−1^. (**c**) Galvanostatic charge–discharge (GCD) curves and (**d**) capacity retention of MoS_2_/FeS_2_ at 0.1 A g^−1^. (**e**) Rate capability and (**f**) corresponding GCD curves from 0.1 to 5.0 A g^−1^. (**g**) Long cycling performance and (**h**) capacity retention of MoS_2_/FeS_2_ at 1.0 A g^−1^.

The lithium-ion storage performance of the MoS_2_/FeS_2_ anode is evaluated by standard half-cell tests. As shown in Figure 3a, the MoS_2_/FeS_2_ anode can be clearly observed within the circle of the first cycle curve. There are two reduction peaks at 0.50 V and 1.26 V, which correspond to the formation of the SEI layer and the conversion of MoS_2_/FeS_2_ to Mo, Fe, and Li_2_S, respectively. In the next cycle, the reduction peak moves to around 1.42 V, and a new peak appears at 2.05 V due to the irreversible phase transition formed by S and Li_2_S. There is an oxidation peak at 1.81 V, which corresponds to the reaction of, Mo, Fe, and Li_2_S to form Li_x_MoS_2_ and Li_x_FeS_2_. There is another oxidation peak at 2.28 V, corresponding to the decomposition of Li_x_MoS_2_ and Li_x_FeS_2_ to MoS_2_ and FeS_2_. As the cycle progresses, the oxidation peak located at 2.28 V undergoes a small shift towards higher voltages. This phenomenon is thought to result from the potential deviation from the equilibrium potential caused by irreversible resistance [30]. The reduction peak at 0.50 V fades away in the next cycle due to the SEI layer forming an anodic surface coating, and the other peaks do not change much, indicating good electrochemical reversibility. The overlapped CV curves imply its outstanding structure and cycling stability. This result is related to efficient covalent bond interaction at the interface of MoS_2_/FeS_2_. Figure 3b shows the cycling performance and Coulombic efficiency of MoS_2_/FeS_2_, pure MoS_2,_ and FeS_2_ at 0.1 A g^−1^. For MoS_2_/FeS_2_, the first discharge-specific capacity is about 1670.6 mA h g^−1^, and the initial Coulombic efficiency (ICE) is ~79.63%. In comparison, the ICE of MoS_2_ is only 73.99%, with a first discharge-specific capacity of 1151.3 mA h g^−1^, and an ICE of FeS_2_ (81.65%); although slightly higher than that of MoS_2_/FeS_2_, it has a first discharge-specific capacity of only 1080.9 mA h g^−1^. Unlike conventional conversion reaction anodes with major structure invalidation and capacity loss, the interfacial engineering strategy can accelerate charge transfer kinetics, adsorption, and intercalation ability. For the MoS_2_/FeS_2_ anode, the discharge capacity can stabilize at 1217.8 mA h g^−1^, and the Coulombic efficiency can be maintained at about 100% after 200 cycles. In contrast, the discharge capacity of the MoS_2_ negative electrode decreased continuously at a current density of 0.1 A g^−1^ and was only 463.1 mA h g^−1^ after 200 cycles. Although the discharge capacity of the FeS_2_ negative electrode recovered during the cycling process, it still decreased to 672.3 mA h g^−1^ after 200 cycles, which was much lower than that of the MoS_2_/FeS_2_ negative electrode. In Figure 3c, the GCD curves show superior electrochemical stability and minimum polarization of the MoS_2_/FeS_2_ anode compared to the pure MoS_2_ and FeS_2_ anodes (Figure S4). This is ascribed to the enhanced interfacial interaction and efficient interatomic electron migration caused by distinct heterointerface structures and covalent chemical bond design [31]. In Figure 3d, the trend regarding discharge capacity change is precisely analyzed by capacity retention. Selecting the first discharge capacity as a check, the discharge capacity retentions of the MoS_2_/FeS_2_ anode are calculated. Meanwhile, the discharge capacity retentions of the pure MoS_2_ and FeS_2_ anodes are also calculated (Figure S5). After 200 cycles, the capacity retention rate of the MoS_2_/FeS_2_ anode is 72.9%, which is much higher than the capacity retention rate of 40.22% for the MoS_2_ anode and 62.2% for the FeS_2_ anode. By comparison, the MoS_2_/FeS_2_ anode exhibits higher and stabler capacity retention, which indicates a more stable lithium-ion storage reaction.

In Figure 3e, the rate capability values of the pure MoS_2_, FeS_2_, and MoS_2_/FeS_2_ anodes are further analyzed from 0.1 to 5.0 A g^−1^. For the MoS_2_/FeS_2_ anode, reversible capacity of 1180.2, 967.3, 773.6, 694.3, 656.5, and 517.1 mA h g^−1^ can be achieved at 0.1, 0.5, 1.0, 2.0, 3.0, and 4.0 A g^−1^, respectively. Notably, even at 5.0 A g^−1^, the discharge capacity can be maintained at 480.9 mA h g^−1^. At 0.1 A g^−1^, the discharge capacity can return to 1169.5 mA h g^−1^ after 80 cycles, showing good structural tolerance and electrochemical reversibility. Rate capability analysis shows good lithium-ion adsorption and diffusion kinetics of the MoS_2_/FeS_2_ anode. In addition, the rate capability values of the pure MoS_2_ and FeS_2_ anodes are much inferior to that of the MoS_2_/FeS_2_ anode. This result can be ascribed to the decomposition of the irreversible electrolytes at low potential and volume expansion during the repeated lithium-ion storage reaction. The excellent reversibility of the half cell is further confirmed by the corresponding GCD curves at different current densities in Figure 3f. The enhanced electronic conductivity and lithium-ion transfer kinetics contribute to its remarkable rate capability.

In Figure 3g, the long cycling capacity is further evaluated at 1.0 A g^−1^. The pure MoS_2_ anode shows the highest discharge capacity as the first cycle progresses, and then the discharge capacity starts to decline. For pure FeS_2_, the discharge capacity reduces to 136.6 mA h g^−1^ after 2000 cycles. For FeS_2_, the discharge capacity of 1000.5 mA h g^−1^ for the first cycle is essentially the same as that of MoS_2_/FeS_2_, but the capacity decays rapidly as the cycle progresses. Although the discharge capacity rebounded at 500–1000 cycles, it slowly decayed in the subsequent cycles and dropped to 158.6 mA h g^−1^ after 2000 cycles. Compared with pure MoS_2_ and FeS_2_ anodes, the MoS_2_/FeS_2_ anode displays outstanding cycle durability at 1.0 A g^−1^. For the MoS_2_/FeS_2_ anode, the first discharge capacity is about 1037.2 mA h g^−1^, and the first charge capacity is 903.7 mA h g^−1^, with a high ICE of 87.13%, which is much higher than that of 55.31% for the MoS_2_ anode and 79.47% for the FeS_2_ anode. The discharge capacity is maintained at 638.9 mA h g^−1^, and the Coulombic efficiency stabilizes at about 100% after 2000 cycles. In Figure 3h, capacity retention analysis is performed to refine the discharge capacity fluctuation of the MoS_2_/FeS_2_ anode, and the capacity retention analysis of the pure MoS_2_ and FeS_2_ anodes is also calculated (Figure S6). At a current density of 1.0 A g^−1^, the capacity retention of MoS_2_/FeS_2_ is at a high level, and, after nearly 2000 cycles, the capacity retention of MoS_2_/FeS_2_ is still 61.78%. The capacity retention of MoS_2_ and FeS_2_ stays at a low level, 16.33% and 15.89%, respectively, after nearly 2000 cycles. Compared with the pure MoS_2_ and FeS_2_ anodes, the cycling durability of MoS_2_/FeS_2_ is the highest. The good capacity retention of the MoS_2_/FeS_2_ anode is ascribed to the distinct heterointerface structural design between MoS_2_ and FeS_2_, and the sufficient heterointerface structure can enhance interatomic electron migration with abundant active sites via covalent chemical bond interaction to improve interfacial charge transfer kinetics. Obviously, the MoS_2_/FeS_2_ anode shows its high-efficiency interfacial interaction and lithium-ion storage performance for potential application.

**Figure 4:**
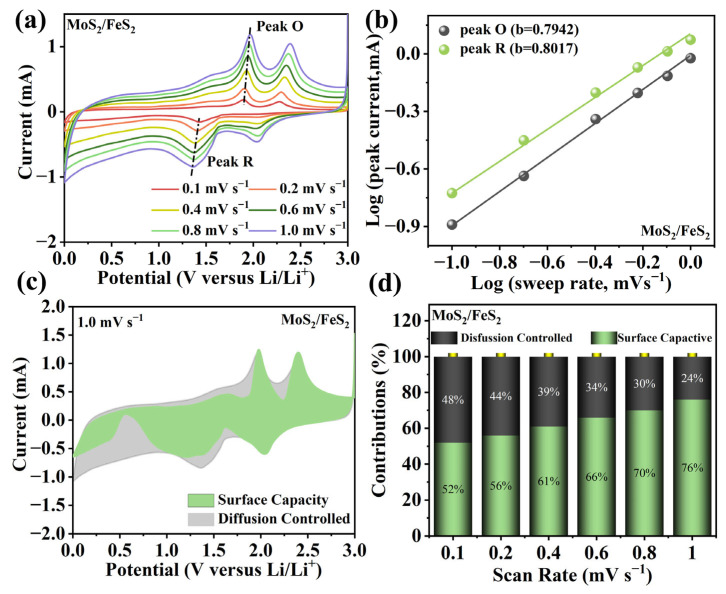
(**a**) CVs analysis, (**b**) *b*-values, (**c**) area ratio image of pseudocapacitance at 1.0 mV s^−1^, and (**d**) pseudocapacitance contribution at different scan rates from 0.1 to 1.0 mV s^−1^ of the MoS_2_/FeS_2_ anodes.

With the scan rate increasing, the oxidation and reduction peaks shift slightly (Figure 4a). The lithium-ion storage capacity is usually composed of diffusion capacity and surface capacity [32]. Meanwhile, qualitative analysis of diffusion capacity and surface capacity can be performed via the following equation:

(1) $$i={av}^{b}$$

where *a* is an adjustable parameter, and the current (*i*) is calculated via the law relationship of sweep rate (*v*) [33]. The *b*-value is the slope of log(*i*) versus log(*v*), which is used to distinguish diffusion capacity (*b* = 0.5) and surface capacity (*b* = 1.0) [34]. In Figure 4b, they are calculated to be 0.7942 and 0.8017, showing the coexistence of diffusion capacity and surface capacity in the MoS_2_/FeS_2_ anode. Quantitative analysis of diffusion capacity and surface capacity can be obtained via the following equation [35]:

(2) $$i\left(V\right)=k_{1}v+k_{2}v^{1/2}$$

At specific potential (*V*), the current (*i*) can be separated as surface capacity (*k*_1_*v*) and diffusion capacity (*k*_2_*v*^1/2^) [36]. In Figure 4c, the pseudocapacitance of the MoS_2_/FeS_2_ anode is as high as 76% at 1.0 mV s^−1^. It is clear that pseudocapacitive behavior dominates the electrochemical processes in the MoS_2_/FeS_2_ heterojunction anode. The distinguished diffusion capacity and surface capacity are shown in Figure 4d. It can be observed that the surface capacity gradually improves, showing the higher surface capacity at high current density. At 0.1 mV s^−1^, the surface capacity is still higher than 50%, resulting in enhanced electrochemical kinetics and excellent rate capability. This pseudocapacitance analysis shows sufficient lithium-ion adsorption in MoS_2_/FeS_2_. Moreover, electrochemical impedance spectra (EIS) analysis is conducted to show the advanced lithium-ion reaction kinetics in the MoS_2_/FeS_2_ anode. In Figure S7, MoS_2_/FeS_2_ and pure MoS_2_ and FeS_2_ anodes have similar EIS curves, which are constituted of a semicircle at medium–high frequency (*R_ct_*) and an inclined line at low frequency (*R_w_*). By comparison, the MoS_2_/FeS_2_ anode exhibits lower charge transfer resistance (*R_ct_* = 80.6 Ω) and lithium-ion transfer resistance than those of the pure MoS_2_ anode (*R_ct_* = 253.4 Ω) and the FeS_2_ anode (*R_ct_* = 335.9 Ω). This suggests that the heterogeneous interface constructed by the in situ hybridization strategy provides low internal resistance and strong lithium-ion migration capability to the MoS_2_/FeS_2_ anode. The improved electrochemical kinetics can be ascribed to the sufficient active sites and the intimate covalent chemical bond interaction.

## 3. Materials and Methods

### 3.1. Synthesis of Precursor

Weigh 0.05 mol Na_2_MoO_4_, add the appropriate amount of deionized water, and stir until completely dissolved. Then, add self-configured 1 M HCl drop by drop until the solution pH = 3, then add 0.05 mol FeCl_3_, stirring well to obtain a golden yellow suspension. It was washed with deionized water and anhydrous ethanol several times, filtered under reduced pressure, and put into a constant temperature vacuum drying oven at 80 °C, and dried for 48 h. After complete drying, the precursor was obtained by grinding.

### 3.2. Synthesis of MoS_2_/FeS_2_ Nanoflower Spheres

Measure 60 mL of deionized water, add 0.15 g of precursor, stirring until completely dispersed. Then, add 0.6 g CH_4_N_2_S and 0.03 g CH_3_(CH_2_)_11_OSO_3_Na, ultrasonic treatment for 1 h until fully mixed, and then add self-configured 1 M NaOH solution drop by drop until the solution pH = 13, and finally transfer to 100 mL PTFE-lined stainless steel kettle, 200 °C hydrothermal reaction for 24 h. After the end of the hydrothermal reaction, cool to room temperature. After washing with deionized water and anhydrous ethanol several times and filtration under reduced pressure, it was put into a constant temperature vacuum drying oven at 60 °C for 48 h. After complete drying, it was milled to obtain the MoS_2_/FeS_2_ nanoflower spheres. To avoid oxidation as much as possible, the final product MoS_2_/FeS_2_ nanoflower spheres are stored in a vacuum glove box.

## 4. Conclusions

In conclusion, a MoS_2_/FeS_2_ interface was successfully constructed via a simple hydrothermal reaction. This well-designed MoS_2_/FeS_2_ efficiently combines interatomic electron migration and lithium-ion adsorption with enhanced charge transfer kinetics. Electrochemical analysis also confirmed the enhanced charge transfer rate and reduced reaction resistance of the MoS_2_/FeS_2_ anode. Benefiting from this interfacial engineering strategy, the MoS_2_/FeS_2_ anode shows a superior cycling capacity of 1180.2 mA h g^−1^ at 0.1 A g^−1^ and a rate capacity of 480.9 mA h g^−1^ at 5.0 A g^−1^. After 200 cycles at 0.1 A g^−1^, the discharge capacity is 1217.8 mA h g^−1^, revealing its good cycling stability. After 2000 cycles at 1.0 A g^−1^, the specific capacity is 638.9 mA h g^−1^ with capacity fading rates of about 0.019% per cycle, respectively, and the corresponding Coulombic efficiency is close to 100%. Overall, the interfacial structural design strategy employed in this study yields a structurally stable MoS_2_/FeS_2_ anode with superior lithium-ion storage performance.

## Data Availability

Data are contained within the article and Supplementary Materials.

## Supplementary Materials

The following supporting information can be downloaded at https://www.mdpi.com/article/10.3390/molecules29061387/s1, Figure S1: SEM of MoS_2_/FeS_2_ ex-situ growth mechanism; Figure S2: SEM of pure (a,b) MoS_2_ and (c,d) FeS_2_; Figure S3: Selected Area Electron Diffraction (SAED) of MoS_2_/FeS_2_; Figure S4: Galvanostatic charge-discharge (GCD) curves of pure MoS_2_ and FeS_2_ at 0.1 A g^−1^; Figure S5: Capacity retention of pure MoS_2_ and FeS_2_ at 0.1 A g^−1^; Figure S6: Capacity retention of pure MoS_2_ and FeS_2_ at 1.0 A g^−1^; Figure S7: EIS of MoS_2_/FeS_2_, pure MoS_2_ and FeS_2_.
