# Supplementary material for: In Situ Hybridization Strategy Constructs Heterogeneous Interfaces to Form Electronically Modulated MoS2/FeS2 as the Anode for High-Performance Lithium-Ion Storage
Source: Molecules. 2024 Mar 20;29(6):1387. doi: 10.3390/molecules29061387 (PMC10975032; doi:10.3390/molecules29061387)
Supplement: Supplementary file 1 [file molecules-29-01387-s001.zip › molecules-2908601-supplementary.pdf]

In-situ hybridization strategy construct heterogeneous interfaces to form electronically modulated MoS_2/FeS_2 as the anode for high performance lithium-ion storage

Dazhi Li[1], Changlong Sun[1], Zeqing Miao[2], Kesheng Gao[3], Zeyang Li[1], Wei Sun[1], Shengjing Guan[4], Xiaofei Qu[1*] and Zhenjiang Li[1*]

[1] *College of Materials Science and Engineering, Qingdao University of Science and Technology, Qingdao 266042, Shandong, P. R. China*

[2] *College of Electromechanical Engineering, Qingdao University of Science and Technology, Qingdao 266061, Shandong, P. R. China*

[3] *Songshan Lake Materials Laboratory, Dongguan 523808, Guangdong, China*

[4] *School of Chemistry and Chemical Engineering, Shandong University of Technology, Zibo 255049, Shandong, China*

*E-mail address:

zhenjiangli@qust.edu.cn (Zhenjiang Li)

quxiaofei@qust.edu.cn (Xiaofei Qu)

Contents:

S1. Characterization methods and details analysis

S2. Results and discussion

Figure S1. SEM of MoS_2/FeS_2 *ex-situ* growth mechanism.

Figure S2. SEM of pure (a-b) MoS_2 and (c-d) FeS_2.

Figure S3. Selected Area Electron Diffraction (SAED) of MoS_2/FeS_2.

Figure S4 Galvanostatic charge-discharge (GCD) curves of pure MoS_2 and FeS_2 at 0.1 A g^{-1}.

Figure S5. Capacity retention of pure MoS_2 and FeS_2 at 0.1 A g^{-1}.

Figure S6. Capacity retention of pure MoS_2 and FeS_2 at 1.0 A g^{-1}.

Figure S7. EIS of MoS_2/FeS_2, pure MoS_2 and FeS_2.

S1. Characterization methods and details analysis

1.1. Materials characterization

The structure and morphology of MoS_2/FeS_2 were characterized by scanning electron microscopy (SEM, Hitachi S-4800). The EDX elemental spectroscopy of pristine MoS_2/FeS_2 was observed using a Talos TEM (FEI, Talos F200X). The powder X-ray diffraction (XRD) measurements were performed on a Rigaku D/MAXRB diffractometer from 5° to 80° (Philips, X'pert Pro MPD, Netherlands), and the excitation wavelength is Cu *Kα* radiation (λ = 0.15 nm). Transmission electron microscopy (TEM) and corresponding high-resolution images were obtained using a Philips Tecnai 20U-Twin microscope at an acceleration voltage of 200 kV. The binding energies of the MoS_2/FeS_2 nanoparticles were acquired by X-ray photoelectron spectroscopy (XPS) on the Thermo ESCALAB 250 with Al Kα radiation (1486.8 eV) as the excitation source. The data fitting was conducted using XPSPEAK 4.1 software. All the XPS spectra were calibrated with the binding energy of C 1s electron at 284.8 eV.

1.2. Device assembly measurements

CR2032-type coin cells were used to investigate the electrochemical performance of MoS_2/FeS_2, pure MoS_2 and FeS_2. The concentration of moisture and oxygen of argon filled glove box are both below 1 ppm. 80 wt% active materials, 10 wt% conductive carbon (ketjen black), and 10 wt% polyvinylidene fluoride (PVDF) as the binder was mixed in N-methyl-2-pyrrolidone, then the mixture was pasted on copper foil and dried at 80 °C. The average loading density of these active materials is 2.0 mg. Li metal is used as both the counter and reference electrodes. Li metal cathode and MoS_2/FeS_2, pure MoS_2 and FeS_2 anodes were electronically separated by a polypropylene film (Celgard 2320) saturated with electrolyte. The electrolyte solution is consist of $LiPF_6$ (1 M) in ethylene carbonate/dimethyl carbonate/diethy carbonate (1:1:1 vol%). Neware CT-3008W system (Neware Technology Ltd., P. R. China) is used to carry out galvanostatic charge and discharge tests at various current densities. The cell was

discharged at 0.5 A g^{-1} for 5 min, followed by a 20 min relaxing from 0.01 to 3.0 V (Shanghai CH Instruments Co., China). Electrochemical impedance spectroscopy (EIS) test is also recorded by CHI660E electrochemical workstation with the frequency ranging from 0.01 Hz to 1 MHz at a 5 mV amplitude signal without applied voltage bias.

S2. Results and discussion

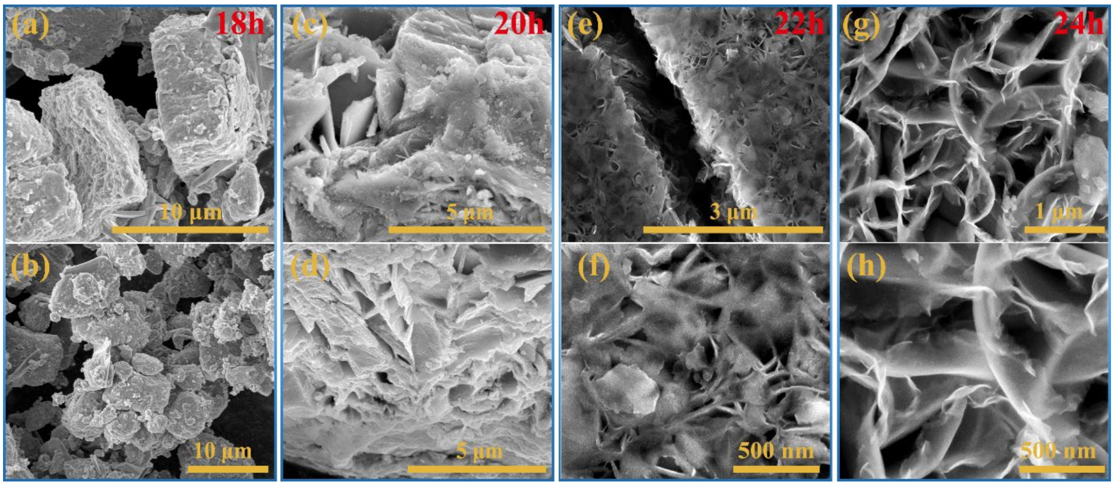

Figure S1. SEM of MoS_2/FeS_2 *ex-situ* growth mechanism.

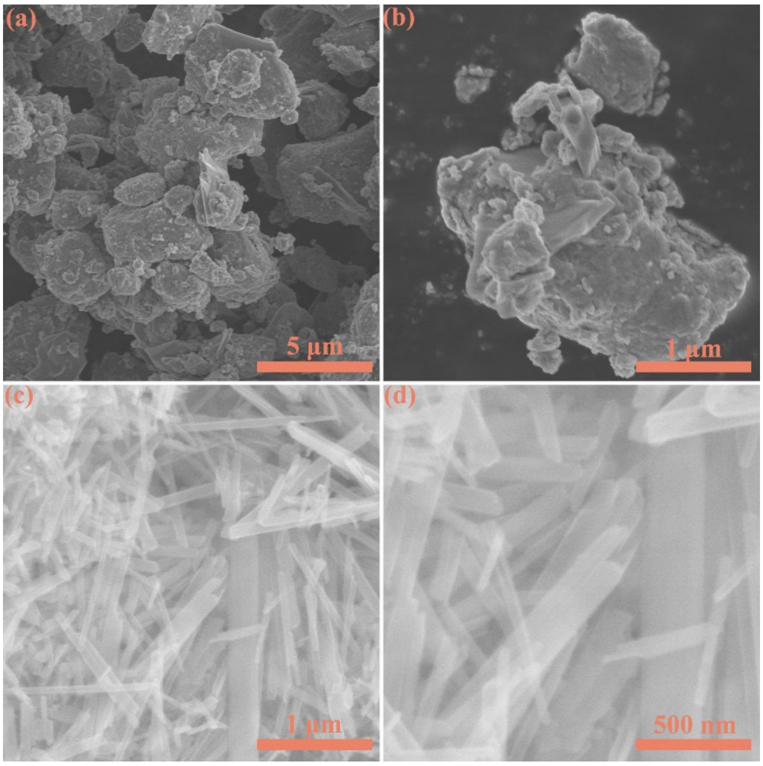

Figure S2. SEM of pure (a-b) MoS_2 and (c-d) FeS_2.

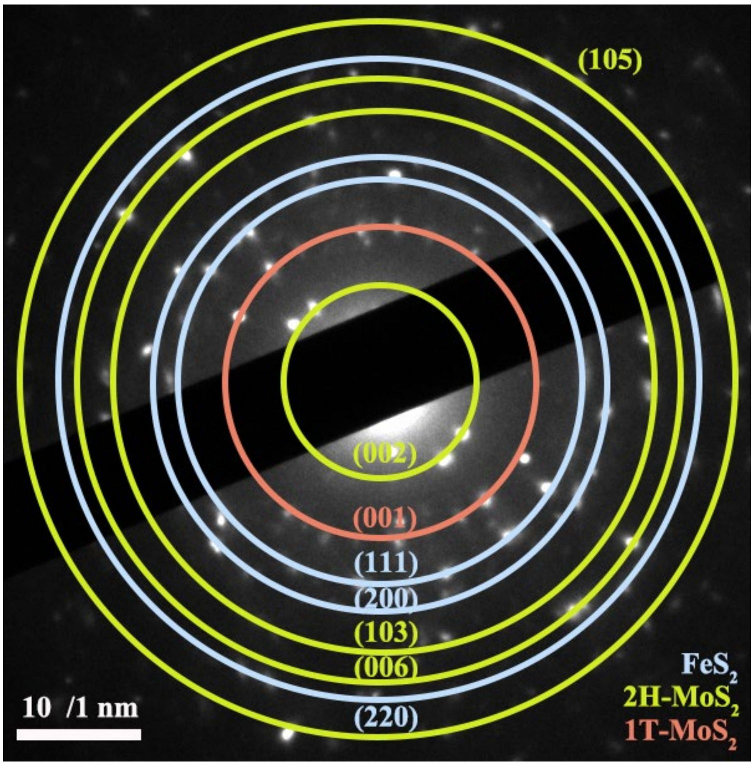

Figure S3. Selected Area Electron Diffraction (SAED) of MoS_2/FeS_2.

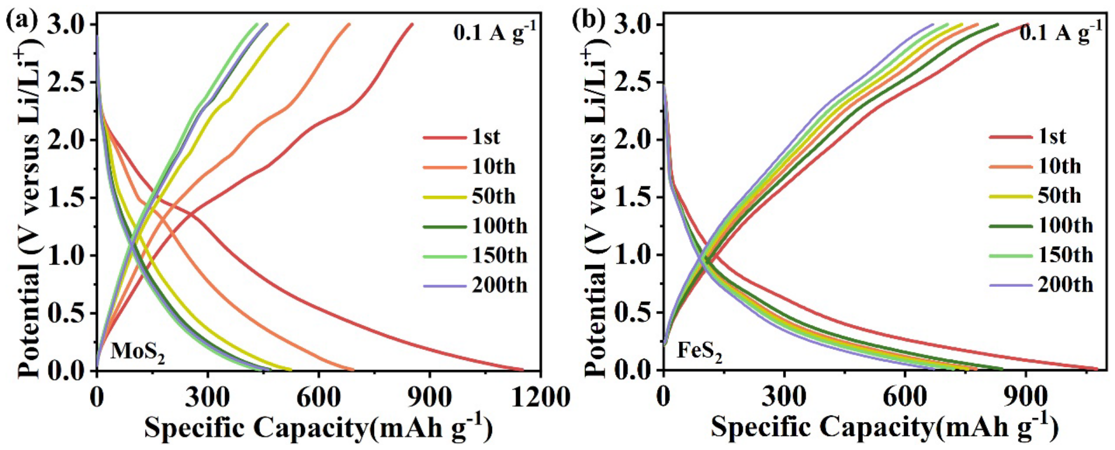

Figure S4 Galvanostatic charge-discharge (GCD) curves of pure MoS_2 and FeS_2 at 0.1 A g^{-1}.

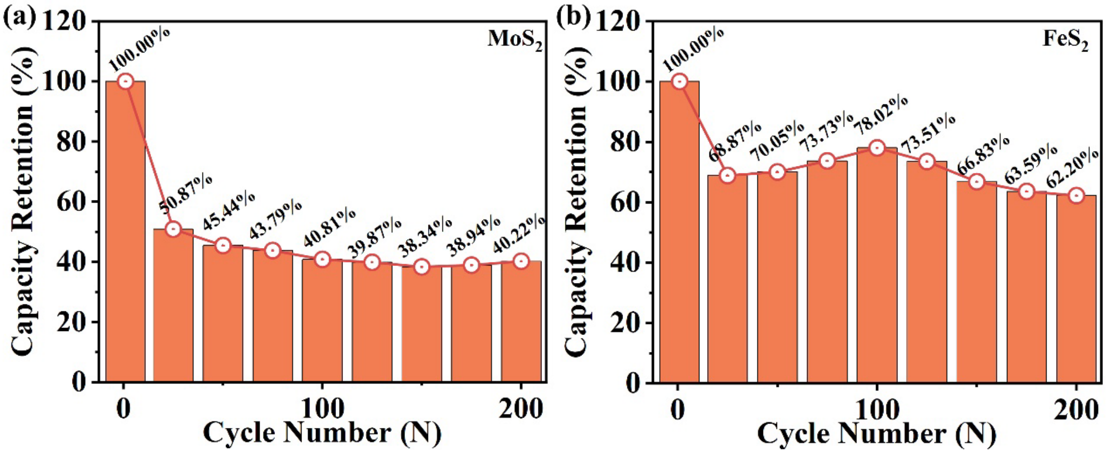

Figure S5. Capacity retention of pure MoS_2 and FeS_2 at 0.1 A g^{-1}.

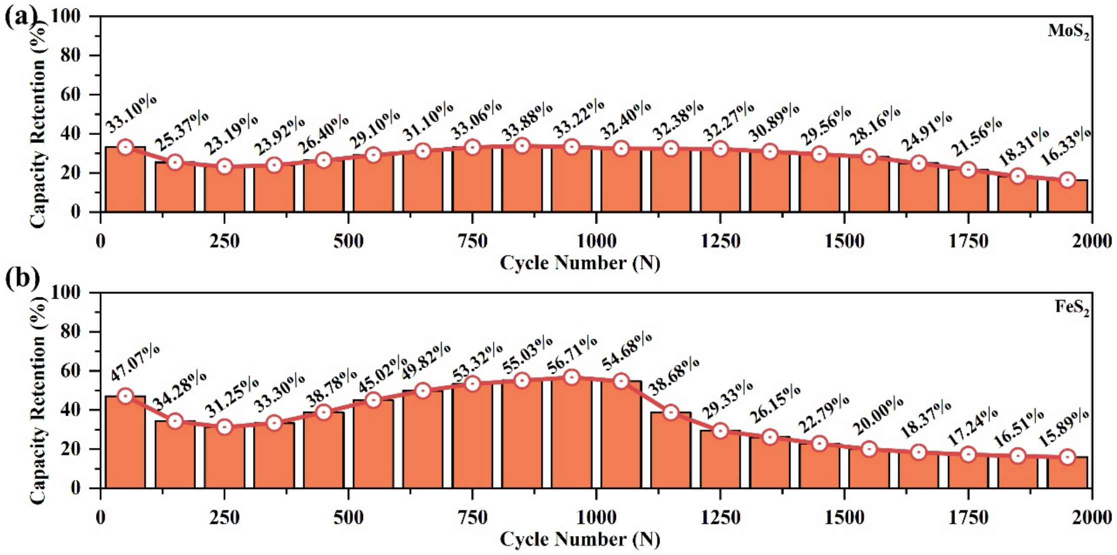

Figure S6. Capacity retention of pure MoS_2 and FeS_2 at 1.0 A g^{-1}.

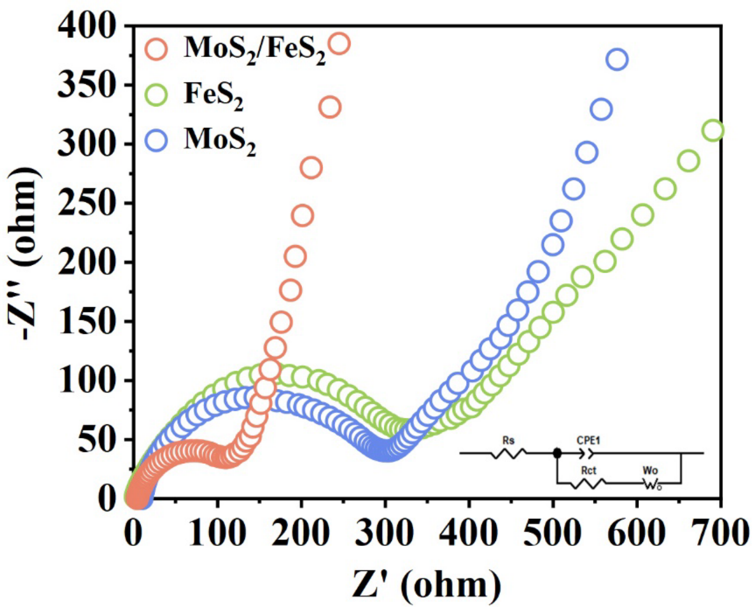

Figure S7. EIS of MoS_2/FeS_2, pure MoS_2 and FeS_2.
